# Reduced Mucosal Associated Invariant T-Cells Are Associated with Increased Disease Severity and *Pseudomonas aeruginosa* Infection in Cystic Fibrosis

**DOI:** 10.1371/journal.pone.0109891

**Published:** 2014-10-08

**Authors:** Daniel J. Smith, Geoffrey R. Hill, Scott C. Bell, David W. Reid

**Affiliations:** 1 Department of Thoracic Medicine, The Prince Charles Hospital, Brisbane, Queensland, Australia; 2 School of Medicine, University of Queensland, Brisbane, Queensland, Australia; 3 Bone Marrow Transplant Laboratory, QIMR Berghofer Medical Research Institute, Brisbane, Queensland, Australia; 4 Infection and Inflammation Laboratory, QIMR Berghofer Medical Research Institute, Brisbane, Queensland, Australia; 5 Queensland Children’s Medical Research Institute, Royal Children’s Hospital, Brisbane, Queensland, Australia; Karolinska Institutet, Sweden

## Abstract

**Background:**

Primary defects in host immune responses have been hypothesised to contribute towards an inability of subjects with cystic fibrosis (CF) to effectively clear pulmonary infections. Innate T-lymphocytes provide rapid pathogen-specific responses prior to the development of classical MHC class I and II restricted T-cell responses and are essential to the initial control of pulmonary infection. We aimed to examine the relationship between peripheral blood lymphocyte phenotype and clinical outcomes in adults with CF.

**Methods:**

We studied 41 subjects with CF and 22, age matched, non-smoking healthy control subjects. Lymphocytes were extracted from peripheral blood samples and phenotyped by flow-cytometry. Lymphocyte phenotype was correlated with sputum microbiology and clinical parameters.

**Results:**

In comparison to healthy control subjects, mucosal associated invariant T (MAIT)-lymphocytes were significantly reduced in the peripheral blood of subjects with CF (1.1% versus 2.0% of T-lymphocytes, P = 0.002). MAIT cell concentration was lowest in CF subjects infected with *P. aeruginosa* and in subjects receiving treatment for a pulmonary exacerbation. Furthermore a reduced MAIT cell concentration correlated with severity of lung disease.

**Conclusion:**

Reduced numbers of MAIT cells in subjects with CF were associated with *P. aeruginosa* pulmonary infection, pulmonary exacerbations and more severe lung disease. These findings provide the impetus for future studies examining the utility of MAIT cells in immunotherapies and vaccine development. Longitudinal studies of MAIT cells as biomarkers of CF pulmonary infection are awaited.

## Introduction

Cystic fibrosis (CF) pulmonary disease is typified by a vicious cycle of bacterial infection and exuberant, but ineffective host immune response [1]. The inability of the intense inflammatory response to clear infection has led to speculation that intrinsic immune defects may contribute to the persistence of pathogens in CF [2]. At the level of the airway lumen, the cellular immune response is dominated by activated neutrophils. However, in contrast, airway epithelial biopsies demonstrate a profound T lymphocyte (T-cell) infiltrate, supporting an important role for adaptive immune responses in the orchestration of a sustained inflammatory response [3].

To date, studies of peripheral, adaptive immune responses in CF have largely focused on the classic dichotomy of T-helper (Th)-1 and Th-2 responses [4]. These early studies suggested a skew towards a Th2 in most CF subjects with *P. aeruginosa* infection, which resulted in increased pulmonary inflammation and disease progression [4]–[6]. The activation of the “classical” adaptive immune response involves antigen recognition, followed by T-cell recruitment and clonal expansion at the site of infection. Consequently, there is a time lag between the host’s recognition of the presence of a pathogen and the development of an effective, adaptive immune response. In recent times, an increasing number of unconventional “innate” T-cell populations have been described (including γ/δ, semi-invariant natural killer (iNKT) and M3-restricted T-cells), which are capable of mounting a more immediate response to pathogens than was previously thought possible. Mucosal associated invariant T (MAIT) cells are a recently described sub-class of innate T-cells, which can be differentiated from other T-cells by the presence of an evolutionary conserved T-cell receptor (TCR) (Vα7.2−Jα33). MAIT cells recognise bacterial and fungal metabolites presented on the major histocompatibility complex (MHC) related protein-1 (MR1) (including the common CF pathogens *Pseudomonas aeruginosa* and *Staphylococcus aureus*), but not viruses [7], [8]. These “innate” T-cell populations provide rapid pathogen-specific responses prior to the development of classical MHC class I and II restricted T-cell responses and importantly may also provide a sustained cytokine response in chronic infection [9], [10].

To date, there is limited knowledge of how changes in circulating lymphocyte populations may relate to pulmonary infection in CF [11]. In this study we performed extensive phenotyping of peripheral blood mononuclear cell populations (PBMCs) obtained from subjects with CF and compared these profiles to those in healthy, age matched, controls. Our particular focus was on the correlation of γ/δ and MAIT innate T-cell values with clinical and microbiological parameters.

## Methods

### Participants and sample collection

Forty-one subjects with CF attending the Adult CF Centre, The Prince Charles Hospital, Queensland, Australia and 22 age-matched, non-smoking, healthy control subjects each supplied a single venous blood sample.

In subjects with CF, total white cell count (WCC), C-reactive protein (CRP) and clinical demographics including, age, CF genotype, lung function, body mass index, pulmonary exacerbation frequency and pulmonary pathogens (based on standard sputum microbiological testing) were recorded. Longitudinal rate of decline in forced expiratory volume in one second (FEV_1_) was determined in CF subjects by means of linear regression analysis (limited to subjects with at least five FEV_1_ measurements recorded over a minimum surveillance period of 2 years).

To explore the effect of pulmonary exacerbations on lymphocyte concentrations a sub-set of 13 “stable” CF subjects (stable respiratory symptoms and a CRP <5 mg L^−1^ at time of blood collection) were compared to eight CF subjects in whom blood was collected within 72 hours of admission to hospital for the intravenous antibiotic treatment of a “pulmonary exacerbation”, defined as increased respiratory symptoms (cough, sputum volume or purulence, dyspnoea) (Figure S1 in File S1).

Ethics approval was obtained from The Prince Charles Hospital, Queensland, Australia, Human Research and Ethics Committee (HREC/11/QPCH/36 and HREC2008∶2885) and all subjects provided written, informed consent.

### Separation and storage of peripheral blood mononuclear cells (PBMCs)

Twelve millilitres of venous blood was collected into lithium heparinised tubes and separated into plasma and cellular components. The cellular component was re-suspended in RPMI (Gibco)+2% heat inactivated Foetal Calf Serum (HiFCS) and PBMCs separated by means of Histopaque 1.077 (Sigma-Aldrich) density gradient separation, as per the manufacturer’s protocol. Following separation, PBMCs were washed twice, re-suspended in RPMI and 15% dimethyl sulfoxide (DMSO), gradually frozen to −80°C and transferred to storage in liquid nitrogen for later batch analysis (concentration 5–20×10^6^ cells/ml).

### Flow Cytometry

PBMCs were rewarmed and re-suspended by drop-wise addition of 10 ml of RPMI+2%HiFCS, washed twice and re-suspended in phosphate buffered saline (PBS) with 1% HiFCS (FACS buffer). A cell count was performed and the volume adjusted to obtain a cell concentration of 10×10^6^ cells/ml.

One hundred micro-litre aliquots of cells were incubated with each of two antibody staining panels as follows:

Panel 1 [adapted from [12]]: FITC anti-human CD16, Pacific Blue anti-human CD14, APC anti-human CD1c, Alexa Fluor700 anti-human CD3, APC/Cy7 anti-human HLA-DR, PE/Cy7 anti-human CD56, PE/Cy7 anti-human CD20, Anti-CD8 antibody – PE Texas (Abcam) and V500 anti-CD4 (BD biosciences) were added to 100 µL of cells and incubated in the dark for 15 minutes. Cells were washed twice in 1 ml of FACS buffer and fixation was performed by incubation with 500 µl of Cytofix (BD biosciences) for 10 minutes. Finally, samples were washed and suspended in 300 µl of FACS buffer.

Panel 2: Surface staining was performed by incubation with V500 anti-CD4, FITC anti-TCR Va7.2, PerCP/Cy5.5 anti-CD161, APC/Cy7 anti-CD3, PE anti-TCR γ/δ, PE Texas red Anti-CD8 for 15 minutes at room temperature in the dark. Cells were washed twice in 1 ml of FACS buffer, fixed and resuspended in 300 µl of buffer solution.

Unless stated otherwise, antibodies were obtained for Biolegend, San Diego. Antibody titration was performed to optimise antibody-cell concentration prior to testing.

Sample analysis was performed on a Fortessa IV flow cytometer (BD Biosciences). A lymphocyte gate was set based on forward and side scatter properties and a minimum of 50,000 gated events were capture for each sample.

MAIT cells were defined as CD3^+^/CD4^−^/CD8^+^or−/CD161^+^/TCR Va7.2^+^ [13].

For subjects with CF, automated haemocytometer, absolute lymphocyte counts were obtained and T-cell sub-types were considered as both, absolute numbers of cells per mL of blood and percentage of the whole T-cell population.

Data analysis was performed using Flowjo version 7.6 (Treestar), representative gating plots are available in Figure S2 in File S1.

### Statistical Analysis

Statistical analysis was performed using PASW, version 18 (SPSS Inc. Chicago IL, USA) and Graph-pad Prism, version 6. Between group differences in PBMC populations were examined using student’s t-test or Mann-Whitney U test. Shapiro-Wilk test and q-q plots were used to determine normal distribution of continuous variables. Non-normally distributed continuous variables (WCC, CRP, MAIT cell absolute count and percentage, B-cell count, NKT-cell and NK-cell percentage) were natural logarithm transformed and Pearson’s correlation used to determine relationships between variables. A p-value<0.05 was considered to represent statistical significance.

## Results

The characteristics of subjects with CF and healthy controls are provided in Table 1. Thirty-six of the 41 subjects with CF had chronic pulmonary infection with *P. aeruginosa* (either in isolation or in combination with another CF respiratory pathogen) on routine microbiological cultures. The remaining five subjects did not have *P. aeruginosa* infection on current, or previous sputum cultures (Table S1 in File S1 for complete sputum microbiological data).

**Table 1:** Subject demographics.

	Healthy Controls	Cystic Fibrosis	P value
Sex (Female:Male)	11∶11	17∶24	0.5
Age (years)	26 (25–32)	28 (22–32)	0.9
BMI (kg/metre^2^)	24.6 (21.4–28.3)	23.5 (20.5–25.8)	0.3
FEV_1_ (Litres)	4.1 (3.5–4.7)	2.2 (1.4–2.5)	<0.001
FEV_1_% Predicted (%)	110 (101–120)	58 (37–75)	<0.001
FVC (Litres)	4.9 (4.4–5.7)	3.1 (2.6–4.1)	<0.001
FVC % Predicted (%)	107 (95–112)	76 (58–84)	<0.001
**CFTR Genotype**			
F508del Homozygotes		20	
F508del Heterozygotes		18	
Other mutations		3	
**Sputum Microbiology** *			
*Pseudomonas aeruginosa*		36	
*Staphylococcus aureus*		12	
*Aspergillus fumigatus*		5	
*Haemophilus influenzae*		4	
*Stenotrophomonas maltophilia*		3	
*Chryseobacterium indologenes*		2	
*Scedosporium apiospermum*		2	
*Mycobacterium intracellulare*		1	
*Burkholderia gladioli*		1	
*Achromobacter xylosoxidans*		1	
Methicillin resistant *S. aureus*		1	

Data presented as median (interquartile range).

*Summary data, subjects may have had more than one pathogen isolated in sputum, individual microbiological data available in Table S1 in File S2.

A greater number of subjects in the pulmonary exacerbation group were male, these subjects were also older, with more severe lung disease, when compared to the stable subjects.

Comparison of lymphocyte sub-sets between groups, demonstrated a reduction in the percentage of MAIT cells in subjects with CF, compared to healthy controls (median 1.1% versus 2.0%, p = 0.002), with an accompanying increase in the percentage of γ/δ T-cells (median 10.4% versus 6.4%, p = 0.012). CF subjects also displayed reduced percentages of NK-cells (median 9.5% versus 13.1%, p = 0.013). The percentage of cells in all of the other major lymphocyte sub-sets was similar between groups (Table 2).

**Table 2:** Comparison of lymphocyte sub-sets between CF and healthy control subjects.

Lymphocyte Population (size gated, CD14−)	CF (n = 41)	Non-CF (n = 22)	P-Value
**T-Cells (CD3+CD16−)**	**72.6 (68.1–79.6)**	**74.9 (67.9–79.0)**	0.6
CD4+CD8−	65.2 (56.1–70.5)	65.1 (59.9–71.4)	0.6
CD8+CD4−	25.7 (21.1–32.5)	27.4 (22.7–32.8)	0.6
MAIT Cells (CD161+, TCR Va7.2+)	1.1 (0.4–1.9)	2.0 (1.4–3.1)	**0.002**
γ/δ T-cells (TCR γ/δ+)	10.4 (6.5–13.4)	6.4 (4.6–9.4)	**0.012**
CD4+CD8+	0.3 (0.2–1.0)	0.4 (0.3–1.7)	0.1
CD4−CD8−*	2.1 (0.9–3.3)	1.6 (1.1–2.3)	0.3
**B-Cells (CD3−CD16−CD20+HLA-DR+)**	**9.5 (4.9–14.3)**	**4.8 (3.9–8.7)**	0.1
Non-Resting (CD1c−)	64.6 (57.0–74.4)	71.6 (60.5–75.1)	0.5
Resting (CD1c+)	35.0 (24.8–43.	28.1 (25.0–38.3)	0.4
**NK-Cells (CD3−)**	**9.5 (6.9–12.2)**	**13.1 (8.0–18.0)**	**0.013**
CD16+CD56dim HLA-DR−	75.1 (61.0–86.7)	88.0 (84.7–92.6)	**0.001**
CD16−CD56+HLA-DR−	8.9 (6.1–13.2)	6.1 (4.5–9.1)	**0.016**
CD16−CD56+HLA-DR+	14.4 (5.1–24.1)	4.3 (2.7–9.2)	**0.005**
**CD3+CD16+**	**2.2 (1.1–3.8)**	**1.8 (1.1–3.6)**	0.7
**Contaminants/Undefined**	**3.7 (3.0–5.2)**	**2.4 (1.9–3.9)**	**0.013**

*After exclusion of MAIT and γ/δ T-cells. Values expressed as percentage of parent population, Median (interquartile range). Significance of between group differences determined by Mann-Whitney U test.

### Relationship between MAIT cells, microbiological and clinical parameters in subjects with CF

**Figure 1:**
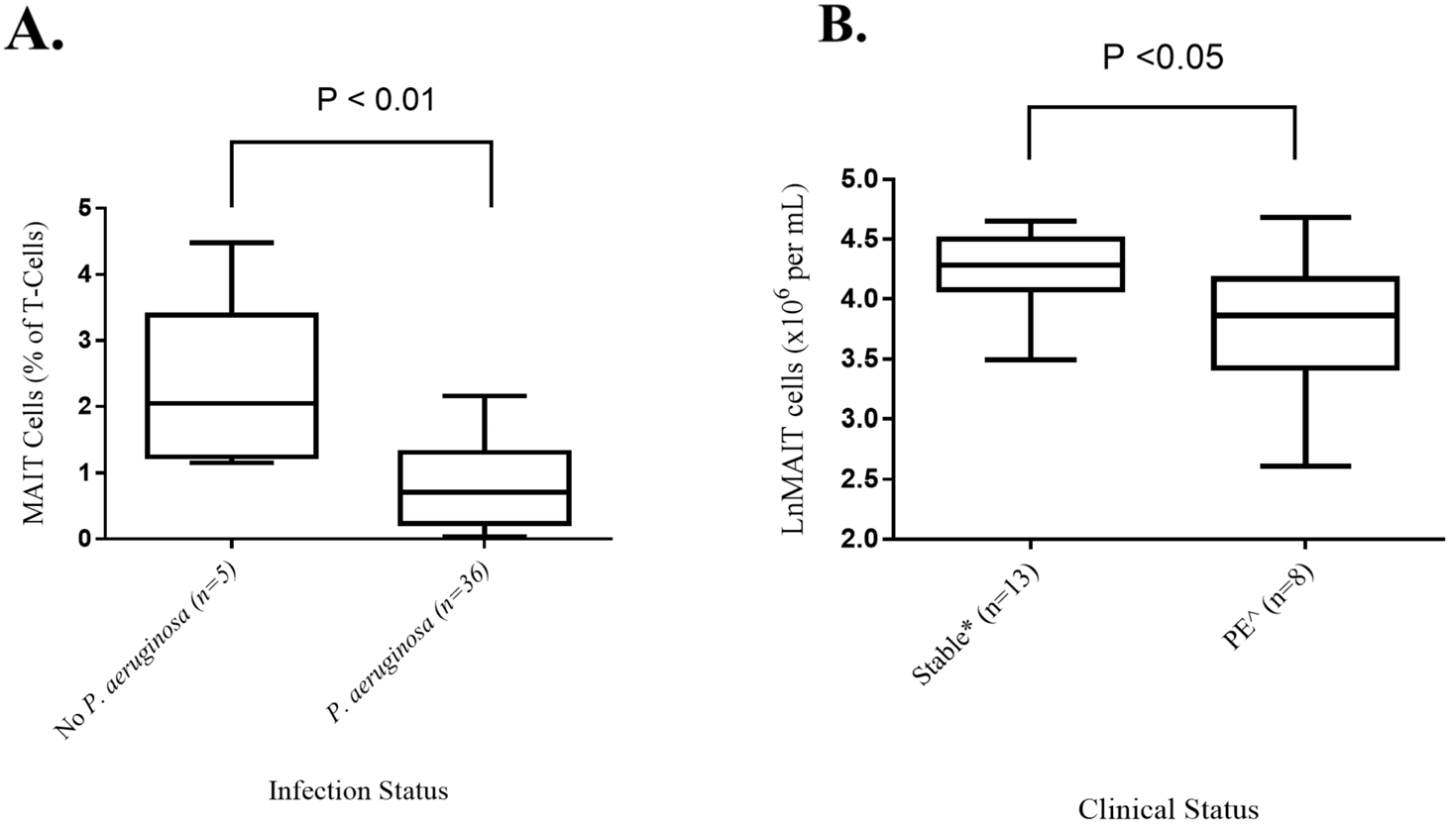
MAIT Cell percentage in CF subjects based on A. Presence of *P. aeruginosa* in sputum cultures, B. Clinical status. PE: pulmonary exacerbations, *two and ∧one not infected with *P. aeruginosa*, between group differences determined by Mann-Whitney U test.

Absolute MAIT cell concentrations and the proportion of T-cells that were MAIT cells (MAIT cell percentage), in the five subjects without *P. aeruginosa* infection were significantly higher than in patients with chronic *P. aeruginosa* infection (Table 3 and Figure 1A). MAIT cell percentages in subjects not infected with *P. aeruginosa* were similar to healthy controls subjects. No difference was seen in the MAIT cell percentage of subjects with a *P. aeruginosa* infection, based on their co-pathogen (Figure S3 in File S1).

**Table 3:** Lymphocyte sub-set phenotypes in CF subjects based on the presence of *P. aeruginosa* infection compared to healthy control subjects.

	CF, No *P. aeruginosa* (n = 5)	CF, *P. aeruginosa* infection (n = 36)	P-Value∧	Non-CF (n = 22)	P-Value^$^
Sex (Female:Male)	3∶2	14∶22	0.4	11∶11	1.0
Age (years)	23.3 (18.4–41.7)	28 (24.5–32.6)	0.8	26 (25–32)	0.5
BMI (kg/metre^2^)	24.7 (19.9–31.7)	23.4 (20.5–25.3)	0.5	24.6 (21.4–28.3)	0.8
FEV_1_% Predicted (%)	72.4 (59.1–88.8)	63.6 (43.4–80.1)	0.3	110 (101–120)	**0.001**
FVC % Predicted (%)	83.9 (68.1–95.9)	79.3 (71.1–88.7)	0.5	107 (95–112)	**0.012**
Lymphocyte Population (size gated, CD14−)					
**T-Cells (CD3+CD16−)**	**68.2 (63.4–76.8)**	**72.9 (68.3–81.3)**	0.4	**74.9 (67.9–79.0)**	0.3
CD4+CD8−	64.4 (49.6–70.0)	65.2 (56.9–70.5)	0.6	65.1 (59.9–71.4)	0.5
CD8+CD4−	26.2 (22.8–38.8)	25.1 (20.5–33.1)	0.6	27.4 (22.7–32.8)	0.9
MAIT Cells (CD161+, TCR Va7.2+)	2.4 (1.4–3.6)	1.0 (0.3–1.8)	**0.023**	2.0 (1.4–3.1)	1.0
γ/δ T-cells (TCR γ/δ+)	8.0 (6.0–16.0)	10.5 (6.6–13.4)	0.7	6.4 (4.6–9.4)	0.2
CD4+CD8+	0.2 (0.2–0.2)	0.3 (0.2–1.1)	**0.014**	0.4 (0.3–1.7)	**0.004**
CD4−CD8−*	3.1 (1.6–4.1)	2.1 (0.7–2.8)	0.2	1.6 (1.1–2.3)	0.1
**B-Cells (CD3−CD16−CD20+HLA-DR+)**	**17.2 (14.0–20.9)**	**8.5 (3.6–13.0)**	**0.002**	**4.8 (3.9–8.7)**	**0.003**
Non-Resting (CD1c−)	74.5 (69.1–79.0)	64.1 (55.5–73.7)	0.1	71.6 (60.5–75.1)	0.1
Resting (CD1c+)	24.9 (20.3–30.5)	35.9 (25.3–43.9)	0.1	28.1 (25.0–38.3)	0.1
**NK-Cells (CD3−)**	**9.6 (4.6–10.9)**	**9.5 (7.8–12.5)**	**0.5**	**13.1 (8.0–18.0)**	**0.039**
CD16+CD56dim HLA-DR−	69.2 (48.5–80.9)	76.1 (63.8–88.1)	0.2	88.0 (84.7–92.6)	**0.006**
CD16−CD56+HLA-DR−	10.9 (7.1–14.5)	8.5 (6.1–13.3)	0.5	6.1 (4.5–9.1)	**0.1**
CD16−CD56+HLA-DR+	21.4 (10.8–37.4)	14.2 (4.5–23.8)	0.2	4.3 (2.7–9.2)	**0.006**
**CD3+CD16+**	**2.0 (0.9–2.6)**	**2.5 (1.0–4.2)**	0.4	**1.8 (1.1–3.6)**	0.9
**Contaminants/Undefined**	**1.8 (1.6–3.5)**	**3.7 (3.1–5.4)**	0.017	**2.4 (1.9–3.9)**	**0.4**

∧CF No *P. aeruginosa versus* CF *P. aeruginosa* infection. ^$^CF No *P. aeruginosa versus* Non-CF. *After exclusion of MAIT and γ/δ T-cells. Values expressed as percentage of parent population, Median (interquartile range). Significance of between group differences determined by Mann-Whitney U test.

Absolute blood MAIT cell counts in the sub-group of stable CF subjects were higher, when compared to subjects sampled early in the course of treatment for a pulmonary exacerbation (Figure 1B). However, the MAIT cell percentage was similar between stable and pulmonary exacerbation subjects (Table 4).

**Table 4:** Lymphocyte sub-sets in CF subjects based on clinical stability and compared to healthy control subjects.

	CF, Pulmonary exacerbation (n = 8)	CF, Stable (n = 13)	P-Value∧	Non-CF (n = 22)	P-Value^$^
Sex (Female:Male)	1∶7	8∶5	**0.027**	11∶11	0.5
Age (years)	32.2 (30.1–39.1)	22.7 (20.5–26.9)	**0.001**	26 (25–32)	**0.010**
BMI (kg/metre^2^)	24.6 (23.5–26.0)	23.0 (21.5–27.0)	0.4	24.6 (21.4–28.3)	0.7
FEV_1_% Predicted (%)	49.7 (40.3–68.5)	83.2 (74.1–88.8)	**0.001**	110 (101–120)	**<0.001**
FVC % Predicted (%)	74.6 (71.2–79.3)	90.0 (85.9–96.4)	**0.001**	107 (95–112)	**0.001**
Lymphocyte Population (size gated, CD14−)					
**T-Cells (CD3+CD16−)**	**71.6 (68.1–82.3)**	**71.3 (67.1–77.1)**	0.6	**74.9 (67.9–79.0)**	0.3
CD4+CD8−	65.9 (52.2–76.2)	58.3 (55.3–68.9)	0.5	65.1 (59.9–71.4)	0.1
CD8+CD4−	23.8 (16.4–37.8)	29.2 (23.6–35.3)	0.3	27.4 (22.7–32.8)	0.7
MAIT Cells (CD161+, TCR Va7.2+)	1.1 (0.3–2.1)	1.1 (0.9–1.9)	0.7	2.0 (1.4–3.1)	**0.018**
γ/δ T-cells (TCR γ/δ+)	11.1 (7.1–16.5)	10.5 (6.4–13.6)	0.8	6.4 (4.6–9.4)	**0.017**
CD4+CD8+	0.2 (0.2–1.0)	0.2 (0.2–0.3)	0.5	0.4 (0.3–1.7)	**0.010**
CD4−CD8−*	2.6 (1.4–3.5)	2.2 (1.3–3.4)	0.6	1.6 (1.1–2.3)	0.1
**B-Cells (CD3−CD16−CD20+HLA-DR+)**	**9.6 (2.5–14.0)**	**13.1 (8.5–16.7)**	0.3	**4.8 (3.9–8.7)**	**0.001**
Non-Resting (CD1c−)	73.4. (65.5–79.9)	70.4 (59.9–79.9)	0.8	71.6 (60.5–75.1)	0.7
Resting (CD1c+)	25.6 (19.9–34.1)	29.3 (18.1–40.1)	0.8	28.1 (25.0–38.3)	0.7
**NK-Cells (CD3−)**	**10.1 (5.9–12.6)**	**9.6 (5.4–11.8)**	0.7	**13.1 (8.0–18.0)**	**0.020**
CD16+CD56dim HLA-DR−	70.7 (52.9–83.7)	69.2 (55.3–80.5)	0.9	88.0 (84.7–92.6)	**<0.001**
CD16−CD56+HLA-DR−	9.9 (8.6–12.6)	8.0 (6.7–13.3)	0.6	6.1 (4.5–9.1)	**0.026**
CD16−CD56+HLA-DR+	19.4 (5.8–37.2)	21.4 (13.2–30.6)	0.8	4.3 (2.7–9.2)	**<0.001**
**CD3+CD16+**	**2.0 (1.2–4.0)**	**2.5 (1.1–4.2)**	1.0	**1.8 (1.1–3.6)**	0.7
**Contaminants/Undefined**	**3.6 (3.1–4.2)**	**3.5 (2.5–4.1)**	0.6	**2.4 (1.9–3.9)**	**0.4**

∧CF pulmonary exacerbation *versus* CF stable, ^$^CF stable *versus* Non-CF. *After exclusion of MAIT and γ/δ T-cells. Values expressed as percentage of parent population, Median (interquartile range). Significance of between group differences determined by Mann-Whitney U test.

**Figure 2:**
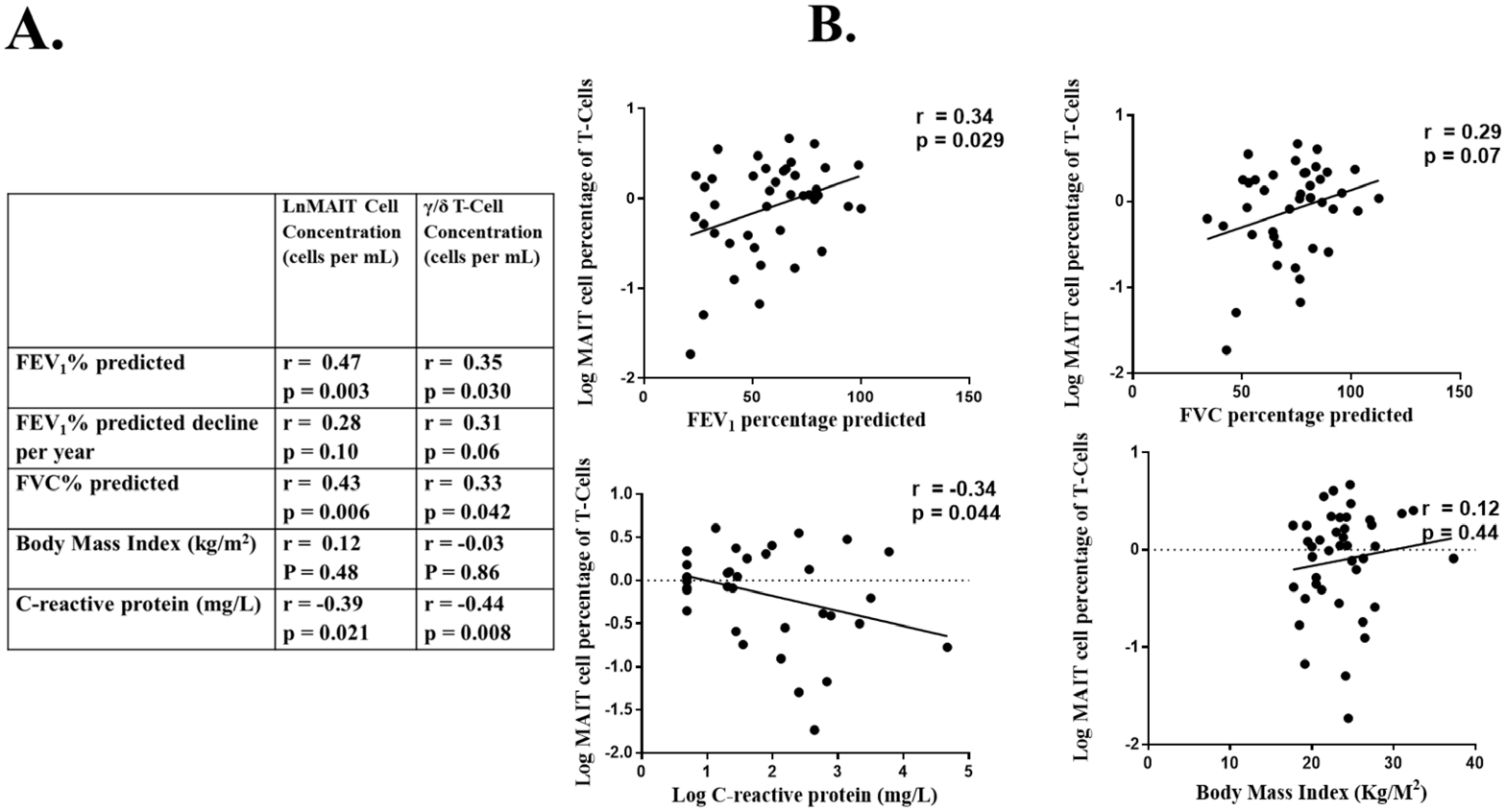
Relationship between MAIT and γ/δ T-cell counts and percentages with lung function. A. Pearson’s Correlation co-efficient (r) and significance value (p) of MAIT and γ/δ T-cells and B. Correlation plots for MAIT Cell expressed as percentage of T-cell population, with FEV_1_ and FVC % predicted, C-reactive protein and body mass index. MAIT: Mucosal invariant T-Lymphocytes, γ/δ T-Cell: Gamma-Delta T-lymphocytes, FEV_1_: Forced expiratory volume in one second, FVC: Forced vital capacity.

Absolute MAIT cell count and MAIT cell percentage correlated positively with FEV_1_ and FVC percentage predicted. A weak relationship was seen between increased rate of FEV_1_ decline and MAIT cells counts, but this did not reach statistical significance (Figure 2).

MAIT cell percentage and absolute MAIT cell concentrations were inversely correlated with CRP (r = −0.34, p = 0.044 and r = −0.39, p = 0.021, respectively), however, there was no relationship between MAIT cells and total WCC.

### Relationship between γ/δ T-cells, microbiological and clinical parameters in subjects with CF

Absolute γ/δ T-cell counts correlated with FEV_1_ and FVC percentage predicted values. A trend toward more rapid rate of lung function decline in FEV_1_ was seen in subjects with lower γ/δ T-cells numbers (Figure 2A). No relationship between γ/δ T-cells when expressed as a percentage of total T-cells and lung function parameters was observed (Figure 2B).

γ/δ T-cell percentage and absolute cell number were inversely correlated with CRP (r = −0.44, p = 0.008) and absolute γ/δ T-cell number was higher in stable CF subjects compared to subjects experiencing a pulmonary exacerbation (Figure S4 in File S1).

There was no relationship between *γ/δ* T-cell counts or percentages and absolute WCC or profile of infection with *P. aeruginosa* (Table 3).

### Relationship between other lymphocyte sub-subset and clinical parameter

Absolute blood lymphocyte count was positively correlated with FEV_1_ and FVC (litres and percentage predicted), however, no relationship was seen between absolute WCC and the percentage of the major lymphocyte sub-sets (T-cells, B-cells, NK-cells, CD3+/CD16+ cells) or lung function (Table S2 in File S1).

Total lymphocyte count was decreased in CF subjects being treated for a pulmonary exacerbation.

B-cell percentage was higher in subjects not infected with *P. aeruginosa*, compared to CF subjects with chronic *P. aeruginosa* infection and healthy controls. There was a trend towards lower B-cell percentages in CF subjects during a pulmonary exacerbation compared to stable CF patients (Figure S5A and B in File S1).

## Discussion

In this study we demonstrate for the first time that the peripheral blood of subjects with CF is characterized by a relative lymphopenia and major reductions in circulating MAIT cells and to a lesser extent an increase in γ/δ T-cells compared to normal healthy controls, consistent with both quantitative and qualitative differences in innate T-cell immunity in CF. Importantly, lung disease severity, systemic inflammation, clinical status and the presence or absence of chronic *P. aeruginosa* infection were all significantly related to the number of circulating MAIT cells in peripheral blood.

The reduction in absolute lymphocyte counts as lung disease severity increases in the current study is consistent with a single previous study of peripheral blood lymphocytes in children with CF [11]. Similar to this earlier study, a reduction in the percentage of NK-cells was also seen in subjects with CF, however, in contrast, no difference in the percentage of CD4+ T cells was seen and the percentages of other major lymphocyte subsets were similar between CF subjects and healthy control subjects [11]. NK-cells represent an innate, cytotoxic subset of lymphocyte which primarily respond to viral infections and tumour cells [14]. In addition, NK cells provide critical support to the eradication of bacterial pulmonary infection (including *P. aeruginosa*), principally through the generation of Th2 cytokines and IFN-γ [15], [16].

A higher percentage of B-cells was seen in CF subjects not infected with *P. aeruginosa* and a trend towards lower concentrations in subjects who were undergoing treatment for an acute pulmonary exacerbation. B-cells are critical for pulmonary protection against encapsulated bacteria and B-cells cultured *in* *vitro* respond to the mucoid exopolysaccharide of *P. aeruginosa* [17]. Further longitudinal studies are required to delineate the role of B-cells during acute pulmonary exacerbations and to determine whether changes in circulating numbers can be attributed to peripheral destruction, tissue sequestration or transformation to plasma cells.

The association between MAIT cells counts and *P. aeruginosa* infection, severe lung function impairment, increased systemic inflammation and acute pulmonary exacerbations in subjects with CF may suggest that MAIT cell deficiency is associated with susceptibility to pulmonary infection in CF. However, the sub-group of CF subjects undergoing a pulmonary exacerbation may have been biased towards a lower MAIT cell concentration, as more subjects in this sub-group were male and in general they had poorer baseline lung function [18]. Furthermore, these data are cross-sectional and do not inform on whether low MAIT cell numbers contribute to *P. aeruginosa* colonisation and disease progression, or whether low numbers simply reflect depletion of MAIT cells in the circulation, because of recruitment to the airway mucosa.

MAIT cells provide a pivotal link between the innate and adaptive immune responses. The semi-invariant T-cell receptor on MAIT cells recognises metabolite derivatives of pathogens (e.g. vitamin B (riboflavin and folic acid)) presented combined to MR-1 [19]. Activated MAIT cells produce high concentrations of pro-inflammatory interleukin (IL)-17 [20], which invokes a potent cascade of cytokines and chemokines (e.g. IL-8 and G-CSF) that promote neutrophil migration into the airways. Interleukin-17 is involved in neutrophil recruitment in CF and high concentrations have been described in sputum and bronchoalveolar lavage fluid of subjects with CF infected with *P. aeruginosa* and also in those patients with severe disease [21]–[23].

Mouse models demonstrate MAIT cell recruitment into the lungs at a very early stage of bacterial infection and sustained MAIT cell responsiveness during the late stages of infection contributes towards an ongoing cytokine response [10]. In mice with selective MAIT cell deficiency the immune response was ineffective in controlling pulmonary infection [10].

Whilst we provide the first description of circulating MAIT cells in CF, understanding of the role of MAIT cells in human disease is limited. A reduction in the proportion of circulating MAIT cells has been reported in subjects with human immunodeficiency virus (HIV), *Mycobacterium tuberculosis* (MTB) and other severe pulmonary infections, and sepsis [7], [8], [24]–[26]. In HIV infection, blood MAIT cells concentrations decrease progressively from time of infection, however, MAIT cell density remains relatively preserved in rectal mucosa, suggesting possible preferential recruitment and thus loss from the circulating pool [27], [28]. Similarly, MTB reactive MAIT cells are enriched in lung lymph of healthy people when compared to matched blood samples [29]. Collectively, the results of these earlier studies and our current work suggest that although blood MAIT cell counts may not reflect tissue concentrations, they may still prove to be a useful, surrogate biomarker of the immune response within the lung [30]. We demonstrate a strong, inverse relationship between MAIT cell counts and CRP and the specificity of MAIT cell responsiveness to bacterial and fungal infections, offers the potential for improved specificity compared to CRP, which will respond to both viruses and non-infective sources of systemic inflammation [31]. Further mechanistic studies of MAIT cells in CF, including assessment in the actual airway will provide novel insights into their role in innate and adaptive immunity in the CF lung, including whether effective vaccines can be developed that boost MAIT cell function to allow eradication of key bacterial pathogens.

γ/δ T-cells represent a minor population of circulating T-cells, which has the capacity to expand rapidly in response to bacterial infection [32]. γ/δ T-and MAIT cells share many similarities; they both produce IL-17 and preferentially migrate to mucosal surfaces from the circulation [33]. In murine pulmonary infection models, γ/δ T-cells rapidly accumulate in the lung, in response to a range bacterial pathogens, where they facilitate the influx of neutrophils and subsequent bacterial clearance [32]. The small increase in the proportion of γ/δ T-cells in the blood of CF subjects in the current study is consistent with findings of a single previous study in CF [34]. However, the relationship between γ/δ T-cells and clinical parameters in CF subjects were only significant when absolute γ/δ T-cell concentrations were considered and these relationships may simply reflect changes in the absolute numbers of T-cells, rather than any implying any specific role for γ/δ T-cell in pathology.

Our current study has several limitations. Firstly, cross-sectional data collection, does not inform whether a causal relationships exists between lymphocytes subsets and clinical parameters. Furthermore, the limited number of patients included in the analysis of stable disease *versus* pulmonary exacerbation, and inclusion of only five patients not infected with *P. aeruginosa* means type I statistical errors are possible. Longitudinal studies which collect blood samples from the same subject before and after the acquisition of *P. aeruginosa,* or during a pulmonary exacerbation and again during a period of stable disease are required. Finally, blood lymphocyte population may not reflect airway populations and studies which correlate lymphocyte concentrations in airway biopsies to those in the blood are anticipated.

## Conclusion

In summary, we describe important differences in the proportions of circulating MAIT and γ/δ T-cell in adult patients with CF, compared to healthy control subjects. Reduced numbers of MAIT cells were associated with *P. aeruginosa* pulmonary infection and more severe lung disease. Our findings provide the impetus for future studies examining the utility of MAIT cells in immunotherapies and vaccine development, and longitudinal studies of MAIT cells as biomarkers of CF pulmonary infection.

## Supporting Information

File S1 **Tables S1, S2, and Figures S1–S5.** Table S1 in File S1. Sputum microbiology of CF subjects. Table S2 in File S1. Relationship between White cell count and lymphocyte subsets, and C-reactive protein, body mass index and lung function. Figure S1 in File S1 Flow diagram of subjects include in sub-group analysis. Figure S2 in File S1 Representative flow-cytometry gating plots. Figure S3 in File S1. MAIT cell percentage in CF subjects with *Pseudomonas aeruginosa* and a co-pathogen in sputum culture. Figure S4 in File S1. Comparison of γ/δ T-cell counts in stable subjects and subjects undergoing antibiotic treatment for a pulmonary exacerbation. Figure S5 in File S1. Comparison of lymphocyte counts and percentage of lymphocyte sub-sets between A. Stable and pulmonary exacerbations B. *P. aeruginosa* infected and non-infected CF subjects.
(DOC)

File S2 **Individual Subject Raw Data.**
(XLS)

## References

[1] Elizur A, Cannon CL, Ferkol TW (2008) Airway inflammation in cystic fibrosis. Chest 133: 489–495. 18252915 10.1378/chest.07-1631

[2] Ratner D, Mueller C (2012) Immune responses in Cystic Fibrosis; are they intrinsically defective? Am J Respir Cell Mol Biol. 10.1165/rcmb.2011-0399RT 22403802

[3] Regamey N, Tsartsali L, Hilliard TN, Fuchs O, Tan HL, et al (2012) Distinct patterns of inflammation in the airway lumen and bronchial mucosa of children with cystic fibrosis. Thorax 67: 164–170. 22008188 10.1136/thoraxjnl-2011-200585

[4] Moser C, Jensen PO (2011) Adaptive Immune Responses and Biofilm Infections. Biofilm Infections: 201–214.

[5] Hartl D, Griese M, Kappler M, Zissel G, Reinhardt D, et al (2006) Pulmonary T(H)2 response in Pseudomonas aeruginosa-infected patients with cystic fibrosis. J Allergy Clin Immunol 117: 204–211. 16387607 10.1016/j.jaci.2005.09.023

[6] Moser C, Kjaergaard S, Pressler T, Kharazmi A, Koch C, et al (2000) The immune response to chronic Pseudomonas aeruginosa lung infection in cystic fibrosis patients is predominantly of the Th2 type. APMIS 108: 329–335. 10937769 10.1034/j.1600-0463.2000.d01-64.x

[7] Gold MC, Lewinsohn DM (2013) Co-dependents: MR1-restricted MAIT cells and their antimicrobial function. Nat Rev Microbiol 11: 14–19. 23178389 10.1038/nrmicro2918

[8] Le Bourhis L, Martin E, Peguillet I, Guihot A, Froux N, et al (2010) Antimicrobial activity of mucosal-associated invariant T cells. Nat Immunol 11: 701–708. 20581831 10.1038/ni.1890

[9] Lanier LL (2013) Shades of grey–the blurring view of innate and adaptive immunity. Nat Rev Immunol 13: 73–74. 23469373 10.1038/nri3389

[10] Meierovics A, Yankelevich WJ, Cowley SC (2013) MAIT cells are critical for optimal mucosal immune responses during in vivo pulmonary bacterial infection. Proc Natl Acad Sci U S A 110: E3119–3128. 23898209 10.1073/pnas.1302799110 PMC3746930

[11] Hausler M, Schweizer K, Biesterfel S, Opladen T, Heimann G (2002) Peripheral decrease and pulmonary homing of CD4+CD45RO+ helper memory T cells in cystic fibrosis. Respir Med 96: 87–94. 11860174 10.1053/rmed.2001.1217

[12] Autissier P, Soulas C, Burdo TH, Williams KC (2010) Evaluation of a 12-color flow cytometry panel to study lymphocyte, monocyte, and dendritic cell subsets in humans. Cytometry A 77: 410–419. 20099249 10.1002/cyto.a.20859 PMC11742174

[13] Martin E, Treiner E, Duban L, Guerri L, Laude H, et al (2009) Stepwise development of MAIT cells in mouse and human. PLoS Biol 7: e54. 19278296 10.1371/journal.pbio.1000054 PMC2653554

[14] Hamerman JA, Ogasawara K, Lanier LL (2005) NK cells in innate immunity. Curr Opin Immunol 17: 29–35. 15653307 10.1016/j.coi.2004.11.001

[15] Culley FJ (2009) Natural killer cells in infection and inflammation of the lung. Immunology 128: 151–163. 19740372 10.1111/j.1365-2567.2009.03167.x PMC2767305

[16] Wesselkamper SC, Eppert BL, Motz GT, Lau GW, Hassett DJ, et al (2008) NKG2D is critical for NK cell activation in host defense against Pseudomonas aeruginosa respiratory infection. J Immunol 181: 5481–5489. 18832705 10.4049/jimmunol.181.8.5481 PMC2567053

[17] Daley L, Pier GB, Liporace JD, Eardley DD (1985) Polyclonal B cell stimulation and interleukin 1 induction by the mucoid exopolysaccharide of Pseudomonas aeruginosa associated with cystic fibrosis. J Immunol 134: 3089–3093. 3920310

[18] Novak J, Dobrovolny J, Novakova L, Kozak T (2014) The decrease in number and change in phenotype of mucosal-associated invariant T cells in the elderly and differences in males and females of reproductive age. Scand J Immunol. 10.1111/sji.12193 24846411

[19] Lopez-Sagaseta J, Dulberger CL, McFedries A, Cushman M, Saghatelian A, et al (2013) MAIT recognition of a stimulatory bacterial antigen bound to MR1. J Immunol 191: 5268–5277. 24108697 10.4049/jimmunol.1301958 PMC3819123

[20] Dusseaux M, Martin E, Serriari N, Peguillet I, Premel V, et al (2011) Human MAIT cells are xenobiotic-resistant, tissue-targeted, CD161hi IL-17-secreting T cells. Blood 117: 1250–1259. 21084709 10.1182/blood-2010-08-303339

[21] McAllister F, Henry A, Kreindler JL, Dubin PJ, Ulrich L, et al (2005) Role of IL-17A, IL-17F, and the IL-17 receptor in regulating growth-related oncogene-alpha and granulocyte colony-stimulating factor in bronchial epithelium: implications for airway inflammation in cystic fibrosis. J Immunol 175: 404–412. 15972674 10.4049/jimmunol.175.1.404 PMC2849297

[22] Tan HL, Regamey N, Brown S, Bush A, Lloyd CM, et al (2011) The Th17 pathway in cystic fibrosis lung disease. Am J Respir Crit Care Med 184: 252–258. 21474644 10.1164/rccm.201102-0236OC PMC3381840

[23] Tiringer K, Treis A, Fucik P, Gona M, Gruber S, et al (2013) A Th17- and Th2-skewed cytokine profile in cystic fibrosis lungs represents a potential risk factor for Pseudomonas aeruginosa infection. Am J Respir Crit Care Med 187: 621–629. 23306544 10.1164/rccm.201206-1150OC

[24] Grimaldi D, Le Bourhis L, Sauneuf B, Dechartres A, Rousseau C, et al (2014) Specific MAIT cell behaviour among innate-like T lymphocytes in critically ill patients with severe infections. Intensive Care Med 40: 192–201. 24322275 10.1007/s00134-013-3163-x

[25] Wong EB, Akilimali NA, Govender P, Sullivan ZA, Cosgrove C, et al (2013) Low levels of peripheral CD161++CD8+ mucosal associated invariant T (MAIT) cells are found in HIV and HIV/TB co-infection. PLoS One 8: e83474. 24391773 10.1371/journal.pone.0083474 PMC3877057

[26] Jiang J, Wang X, An H, Yang B, Cao Z, et al (2014) Mucosal-associated Invariant T-Cell Function Is Modulated by Programmed Death-1 Signaling in Patients with Active Tuberculosis. Am J Respir Crit Care Med 190: 329–339. 24977786 10.1164/rccm.201401-0106OC

[27] Cosgrove C, Ussher JE, Rauch A, Gartner K, Kurioka A, et al (2013) Early and nonreversible decrease of CD161++/MAIT cells in HIV infection. Blood 121: 951–961. 23255555 10.1182/blood-2012-06-436436 PMC3567342

[28] Leeansyah E, Ganesh A, Quigley MF, Sonnerborg A, Andersson J, et al (2013) Activation, exhaustion, and persistent decline of the antimicrobial MR1-restricted MAIT-cell population in chronic HIV-1 infection. Blood 121: 1124–1135. 23243281 10.1182/blood-2012-07-445429 PMC3575756

[29] Gold MC, Cerri S, Smyk-Pearson S, Cansler ME, Vogt TM, et al (2010) Human mucosal associated invariant T cells detect bacterially infected cells. PLoS Biol 8: e1000407. 20613858 10.1371/journal.pbio.1000407 PMC2893946

[30] Shoki AH, Mayer-Hamblett N, Wilcox PG, Sin DD, Quon BS (2013) Systematic review of blood biomarkers in cystic fibrosis pulmonary exacerbations. Chest 144: 1659–1670. 23868694 10.1378/chest.13-0693

[31] Levy H, Kalish LA, Huntington I, Weller N, Gerard C, et al (2007) Inflammatory markers of lung disease in adult patients with cystic fibrosis. Pediatr Pulmonol 42: 256–262. 17245735 10.1002/ppul.20563 PMC4469989

[32] Ito M, Kojiro N, Ikeda T, Ito T, Funada J, et al (1992) Increased proportions of peripheral blood gamma delta T cells in patients with pulmonary tuberculosis. Chest 102: 195–197. 1385570 10.1378/chest.102.1.195

[33] Sutton CE, Mielke LA, Mills KH (2012) IL-17-producing gammadelta T cells and innate lymphoid cells. Eur J Immunol 42: 2221–2231. 22949320 10.1002/eji.201242569

[34] Raga S, Julia MR, Crespi C, Figuerola J, Martinez N, et al (2003) Gammadelta T lymphocytes from cystic fibrosis patients and healthy donors are high TNF-alpha and IFN-gamma-producers in response to Pseudomonas aeruginosa. Respir Res 4: 9. 14525626 10.1186/1465-9921-4-9 PMC203157

