# Supplementary material for: Reduced Mucosal Associated Invariant T-Cells Are Associated with Increased Disease Severity and Pseudomonas aeruginosa Infection in Cystic Fibrosis
Source: PLoS One. 2014 Oct 8;9(10):e109891. doi: 10.1371/journal.pone.0109891 (PMC4190362; doi:10.1371/journal.pone.0109891)
Supplement: File S1 — Tables S1, S2, and Figures S1–S5. Table S1 in File S1. Sputum microbiology of CF subjects. Table S2 in File S1. Relationship between White cell count and lymphocyte subsets, and C-reactive protein, body mass index and lung function. Figure S1 in File S1 Flow diagram of subjects include in sub-group analysis. Figure S2 in File S1 Representative flow-cytometry gating plots. Figure S3 in File S1. MAIT cell percentage in CF subjects with Pseudomonas aeruginosa and a co-pathogen in sputum culture. Figure S4 in File S1. Comparison of γ/δ T-cell counts in stable subjects and subjects undergoing antibiotic treatment for a pulmonary exacerbation. Figure S5 in File S1. Comparison of lymphocyte counts and percentage of lymphocyte sub-sets between A. Stable and pulmonary exacerbations B. P. aeruginosa infected and non-infected CF subjects. (DOC) [file pone.0109891.s001.doc]

**Table S1**. Sputum microbiology of CF subjects

| Subject No. | Gender | Age | FEV1 percentage predicted | Body Mass Index | *Pseudomonas aeruginosa* | *Staphylococcus aureus* | Methicillin resistant *Staphylococcus aureus* | *Achromobacter xylosoxidans* | *Burkholderia gladioli* | *Scedosporium apiospermum* | *Chryseobacterium indologenes* | *Stenotrophomonas maltophilia* | *Aspergillus fumigatus* | *Haemophilus influenae* | *Mycobacteriun avian intracellulare* |
| --- | --- | --- | --- | --- | --- | --- | --- | --- | --- | --- | --- | --- | --- | --- | --- |
| 23 | Male | 29 | 24.0 | 19.4 | ✓ |  |  |  |  |  |  |  |  |  |  |
| 24 | Female | 19 | 73.4 | 20.0 | ✓ |  |  |  |  |  |  |  |  |  |  |
| 25 | Male | 28 | 82.1 | 27.7 | ✓ |  |  |  |  |  |  |  |  |  |  |
| 26 | Male | 27 | 56.2 | 24.2 | ✓ |  |  |  |  |  |  |  | ✓ |  |  |
| 27 | Male | 32 | 27.4 | 24.1 | ✓ |  |  |  |  |  |  | ✓ |  |  |  |
| 28 | Male | 22 | 58.0 | 19.5 | ✓ | ✓ |  |  |  |  |  |  |  |  |  |
| 29 | Female | 32 | 65.6 | 23.4 | ✓ | ✓ |  |  |  |  |  |  |  | ✓ |  |
| 30 | Female | 27 | 62.9 | 20.5 | ✓ |  |  |  |  |  | ✓ |  |  |  |  |
| 31 | Female | 21 | 67.7 | 23.5 | ✓ |  |  |  |  | ✓ |  |  |  |  |  |
| 32 | Male | 35 | 52.5 | 24.8 | ✓ | ✓ |  | ✓ |  |  |  |  |  |  |  |
| 33 | Male | 31 | 21.5 | 24.5 | ✓ |  | ✓ |  |  |  |  |  |  |  |  |
| 34 | Male | 25 | 56.7 | 37.3 | ✓ |  |  |  |  |  |  |  |  | ✓ |  |
| 35 | Female | 34 | 69.7 | 27.3 | ✓ | ✓ |  |  |  |  |  |  |  |  |  |
| 36 | Male | 26 | 83.6 | 22.4 | ✓ |  |  |  |  |  |  |  |  |  |  |
| 37 | Male | 28 | 100.1 | 24.9 | ✓ | ✓ |  |  |  |  |  |  | ✓ |  |  |
| 38 | Male | 26 | 51.0 | 23.3 | ✓ | ✓ |  |  |  |  |  |  |  |  |  |
| 39 | Female | 22 | 79.5 | 21.0 | ✓ |  |  |  |  |  |  |  |  |  |  |
| 40 | Male | 28 | 27.6 | 20.5 | ✓ |  |  |  |  |  | ✓ |  |  |  |  |
| 41 | Male | 24 | 41.6 | 26.5 | ✓ |  |  |  |  |  |  |  |  |  |  |
| 42 | Male | 40 | 23.6 | 25.4 | ✓ |  |  |  |  |  |  |  |  |  |  |
| 43 | Female | 22 | 78.6 | 22.6 | ✓ |  |  |  |  |  |  |  |  |  |  |
| 44 | Female | 33 | 69.5 | 18.5 | ✓ |  |  |  |  |  |  |  |  |  |  |
| 45 | Male | 32 | 80.3 | 27.8 | ✓ |  |  |  |  |  |  |  |  |  |  |
| 46 | Female | 34 | 64.3 | 27.1 | ✓ |  |  |  |  |  |  |  |  |  |  |
| 47 | Male | 38 | 32.6 | 20.0 | ✓ | ✓ |  |  |  |  |  |  |  |  |  |
| 48 | Female | 20 | 94.2 | 26.3 | ✓ | ✓ |  |  |  |  |  |  |  |  |  |
| 49 | Female | 27 | 53.3 | 19.1 | ✓ |  |  |  |  |  |  |  |  |  |  |
| 50 | Male | 31 | 28.0 | 23.8 | ✓ |  |  |  |  |  |  |  |  |  |  |
| 51 | Female | 25 | 76.2 | 24.3 | ✓ | ✓ |  |  |  |  |  |  |  |  |  |
| 52 | Male | 36 | 53.9 | 26.2 | ✓ |  |  |  |  |  |  |  |  |  |  |
| 53 | Male | 20 | 60.7 | 23.0 | ✓ |  |  |  |  |  |  |  | ✓ |  |  |
| 54 | Male | 34 | 31.5 | 24.0 | ✓ | ✓ |  |  |  |  |  | ✓ |  |  |  |
| 55 | Male | 27 | 34.1 | 21.5 | ✓ |  |  |  |  |  |  |  |  |  |  |
| 56 | Male | 30 | 48.0 | 21.2 | ✓ |  |  |  |  |  |  |  |  |  |  |
| 57 | Female | 24 | 39.6 | 19.2 | ✓ |  |  |  |  |  |  |  |  |  |  |
| 58 | Female | 17 | 32.6 | 17.8 |  |  |  |  |  |  |  | ✓ | ✓ |  |  |
| 59 | Male | 43 | 66.9 | 24.7 |  |  |  |  | ✓ |  |  |  |  |  |  |
| 60 | Female | 18 | 50.3 | 17.7 |  |  |  |  |  | ✓ |  |  |  |  | ✓ |
| 61 | Female | 18 | 99.0 | 31.0 |  |  |  |  |  |  |  |  | ✓ | ✓ |  |
| 62 | Male | 23 | 78.7 | 22.1 |  | ✓ |  |  |  |  |  |  |  | ✓ |  |
| 63 | Female | 40 | 67.8 | 32.4 |  | ✓ |  |  |  |  |  |  |  |  |  |

**Table S2. Relationship between White cell count and lymphocyte subsets, and C-reactive protein, body mass index and lung function.**

|  | FEV1 % Predicted | FVC % Predicted | BMI (Kg/m^2^) | CRP (mg/L) |
| --- | --- | --- | --- | --- |
| White Cell Count* (x10^6^ per mL) | r = 0.17 | r = 0.18 | r = -0.14 | r = -0.02 |
|  | p = 0.30 | p = 0.28 | p = 0.38 | p = 0.92 |
| Lymphocyte (x10^6^ per mL) | **r = 0.50** | **r = 0.49** | r = -0.07 | **r = -0.45** |
|  | **p = 0.001** | **p = 0.002** | p = 0.69 | **p = 0.007** |
| T-cell (% of lymphocytes) | r = -0.12 | r = -0.11 | r = -0.15 | r = 0.28 |
|  | p = 0.48 | p = 0.50 | p = 0.0.36 | p = 0.10 |
| B-Cell (% of lymphocytes) | r = 0.27 | r = 0.17 | **r = 0.34** | r = -0.17 |
|  | p = 0.09 | p = 0.30 | **p = 0.031** | p = 0.31 |
| NKT-Cell* (% of lymphocytes) | r = 0.08 | r = 0.13 | r = -0.14 | r = -0.12 |
|  | p = 0.61 | p = 0.43 | p = 0.48 | p = 0.48 |
| NK-Cell* (% of lymphocytes) | r = -0.07 | r = -0.04 | r = -0.10 | r = -0.25 |
|  | p = 0.68 | p = 0.79 | p = 0.53 | p = 0.15 |
| CRP* (mg/L) | **r = -0.54** | **r = -0.51** | r = -0.22 |  |
|  | **p = 0.001** | **p = 0.002** | p = 0.20 |

FEV1: Forced expiratory volume for one second, FVC: Forced vital capacity. *Ln Transformed prior to analysis. Pearson’s correlations (r) and p-value.

**Figure S1: Flow diagram of subjects include in sub-group analysis**

**
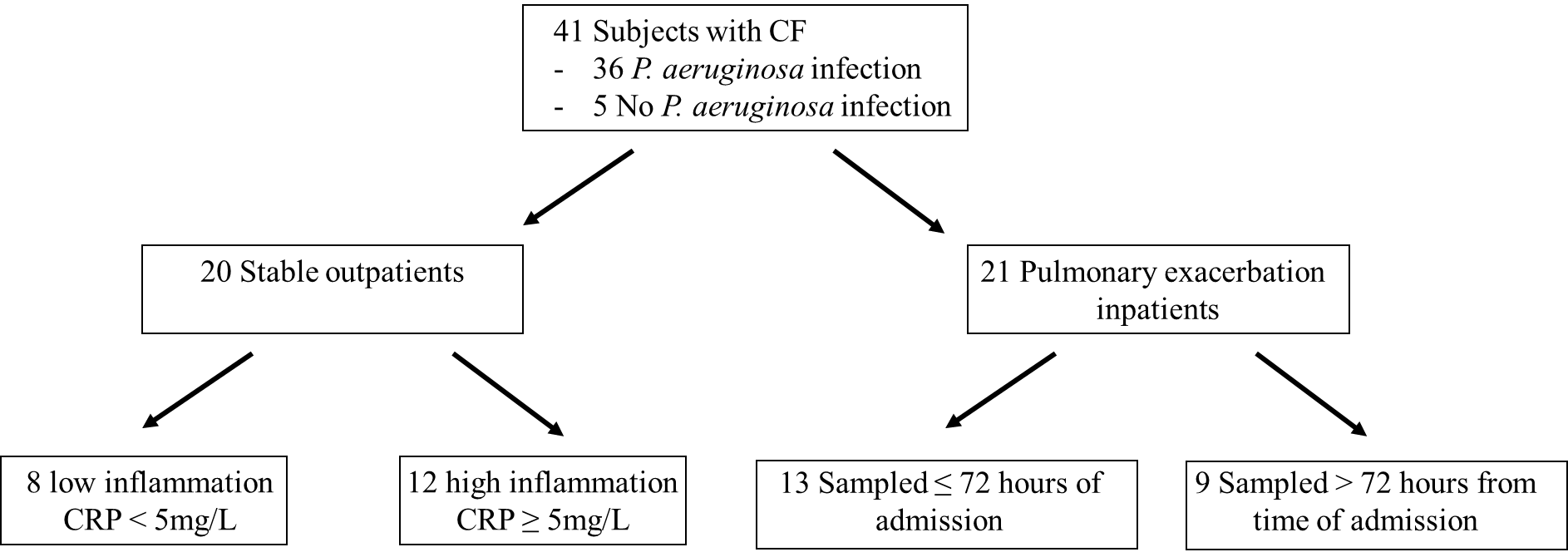
**

**Figure S2: Representative flow-cytometry gating plots.**

Panel 1: Determination of Major Lymphocyte sub-sets.


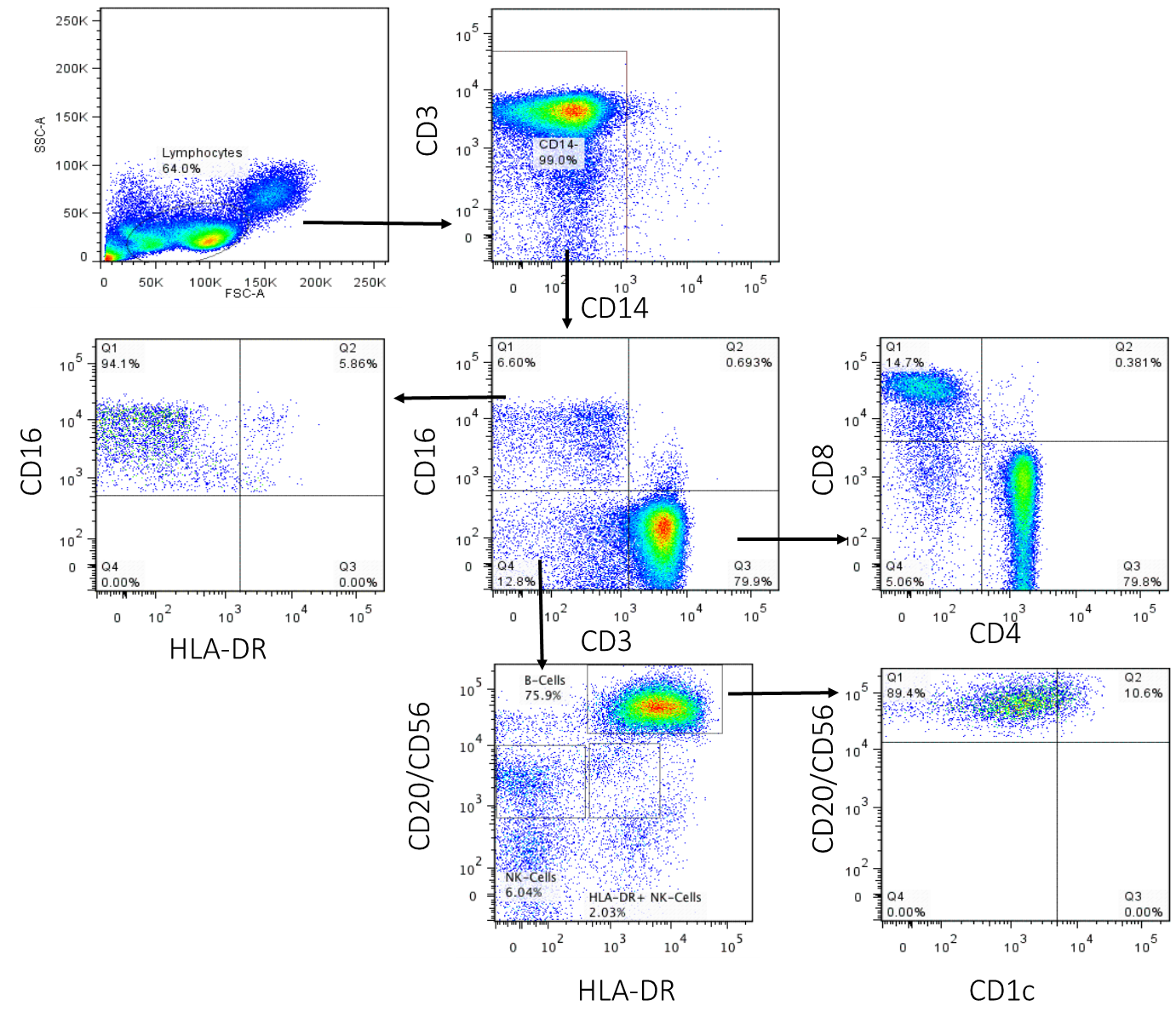


Cell Characteristic; Lymphocytes (Appropriate forward and side scatter properties and CD14-), T-Cells (CD3+/CD16-), CD3+/CD16+ cells, NK-Cells (CD16+/CD3-/HLA-DR-, CD16-/CD3-/CD56+/HLA-DR- and CD16-/CD3-/CD56+/HLA-DR+), CD4+ T-Cells (CD3+/CD16-/CD4+/CD8-), CD8+ T-Cells (CD3+/CD16-/CD4-/CD8+), CD4+/CD8+ T-Cells (CD3+/CD16-/CD4+/CD8+), CD4-/CD8- T-Cells (CD3+/CD16-/CD4-/CD8+), B-Cells (CD3-/CD16-/CD20+/HLA-DR+)

Panel 2: Determination of MAIT cells and γ/δ T-Cells.


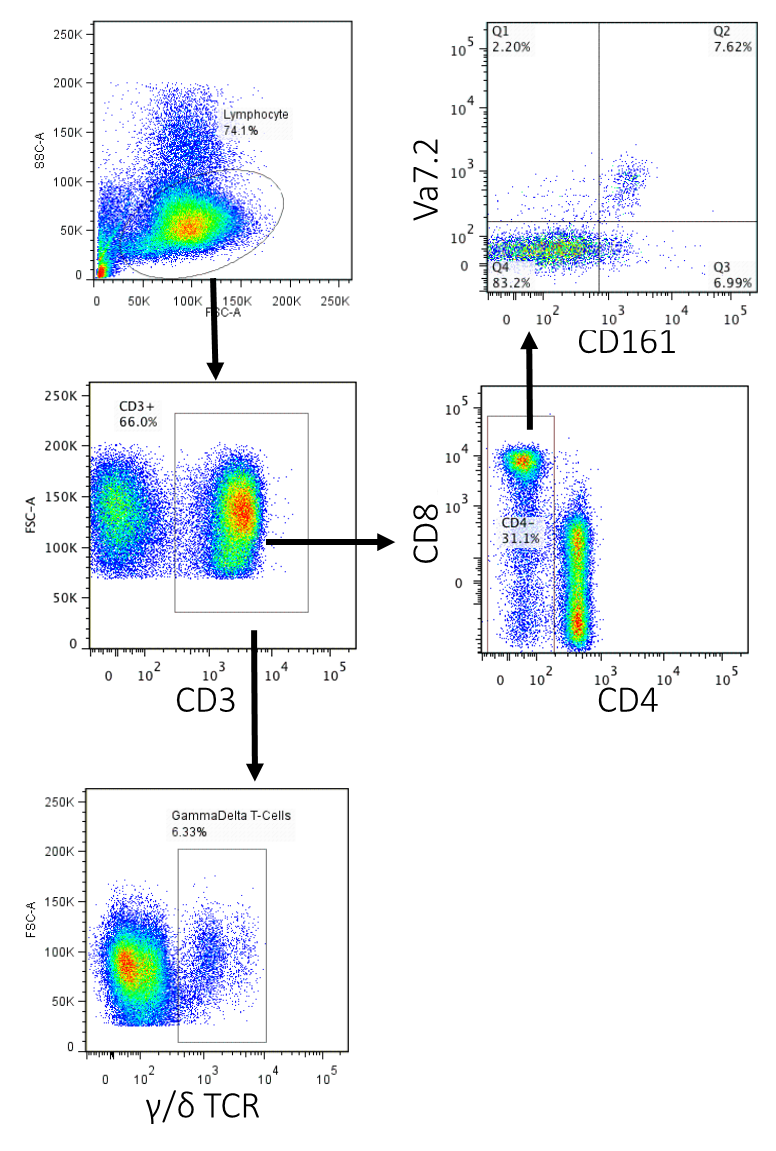


Cell Characteristics: MAIT Cells (CD3+/CD4-/TCR Va7.2+/CD161+), γ/δ T-Cells (CD3+/TCR γ/δ+).

**Figure S3. MAIT cell percentage in CF subjects with *Pseudomonas aeruginosa* and a co-pathogen in sputum culture.**

Individual subjects may be represented multiple times, dependent on the number of co-pathogens isolated from sputum cultures. Horizontal lines represent Median and interquartile range. No significant difference between groups when examined by Kruskal-Wallis test.

**Figure S4. Comparison of γ/δ T-cell counts in stable subjects and subjects undergoing antibiotic treatment for a pulmonary exacerbation.**

PE: pulmonary exacerbations. Between group differences determined by Mann-Whitney U test.

**Figure S5. Comparison of lymphocyte counts and percentage of lymphocyte sub-sets between A. Stable and pulmonary exacerbations B. *P. aeruginosa* infected and non-infected CF subjects.**

**
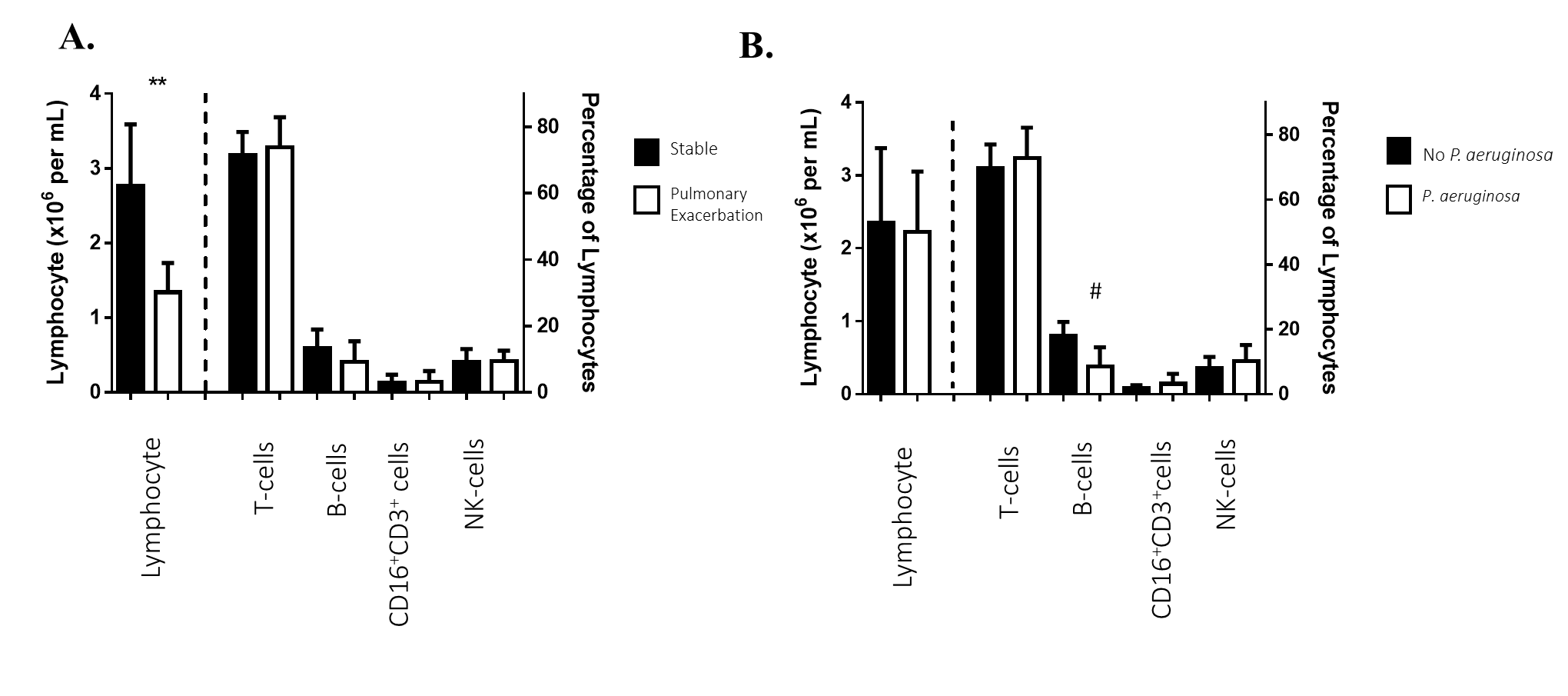
**

Between group differences examined by Mann-Whitney U test. ** P=0.001 ^#^ P=0.004
